# Amplification of Electronic Circular Dichroism—A Tool to Follow Self-Assembly of Chiral Molecular Capsules †

**DOI:** 10.3390/molecules26237100

**Published:** 2021-11-24

**Authors:** Marek P. Szymański, Marcin Grajda, Agnieszka Szumna

**Affiliations:** Institute of Organic Chemistry, Polish Academy of Sciences, Kasprzaka 44/52, 01-224 Warsaw, Poland; mszymanski@icho.edu.pl (M.P.S.); mgrajda@icho.edu.pl (M.G.)

**Keywords:** circular dichroism, self-assembly, molecular capsules, peptides, inherent chirality, DFT calculations

## Abstract

Electronic circular dichroism (ECD) can be used to study various aspects of self-assembly (definition of stoichiometric ratios, chirality amplification during self-assembly, host-guest complexation). In this work, we show that ECD is a valuable tool for monitoring the self-assembly of chiral peptide-based capsules. By analyzing the signs, intensities, and temperature dependences of ECD bands, the effects of the non-specific processes can be separated from the restriction of intramolecular motion (RIM) caused by discrete self-assembly. Analysis of experimental and theoretical ECD spectra show that the differences between assembled and non-assembled species originate from induction of inherently chiral conformation and restriction of conformational freedom that leads to amplification of ECD signals during self-assembly of discrete species.

## 1. Introduction

Self-assembly of molecules under given conditions is a crucial feature that determines their properties and function. Numerous methods of following this process have been elaborated and constitute workhorses of supramolecular chemistry and molecular biology [1]. In principle, any change in physical or spectroscopic property can be used to follow the process of self-assembly. Despite the apparent abundance of methods, in many cases, the unambiguous determination of details of a self-assembly process is not trivial. Especially problematic are the cases of multivalent binding involving high symmetry molecules, which undergo complex equilibria for which the effects of specific and non-specific aggregation cannot be easily separated. Electronic circular dichroism (ECD) is not the most common method to study self-assembly, but numerous studies have demonstrated that it can address various problems, ranging from the definition of simple stoichiometric ratios between the partners, giving rise to a supramolecular structure to the derivation of thermodynamic parameters, or ultimately to the refinement of a structure for a complex or an aggregate [2,3,4,5]. The ECD technique has been found particularly useful for the determination of chirality amplification during self-assembly of supramolecular polymers (according to the “sergeants-and-soldiers” rule) [6,7] and for studying host–guest complexation by induced chirality effects [8]. Despite numerous advantages, the information encoded in ECD spectra is typically difficult to be assessed directly because the ECD spectra include the contributions of all conformers populated at the working temperature [9,10], and prediction of the spectrum even for a single conformer typically requires quantum calculation. In this work, we demonstrate the application of ECD spectroscopy for the monitoring of the self-assembly of chiral peptide-based capsules. We show that by analyzing the signs, intensities, and also temperature dependences of ECD bands, the effects of the non-specific processes (temperature-dependent conformational dynamics) can be separated from the restriction of intramolecular motion (RIM) caused by discrete self-assembly.

## 2. Results and Discussion

**Figure 1:**
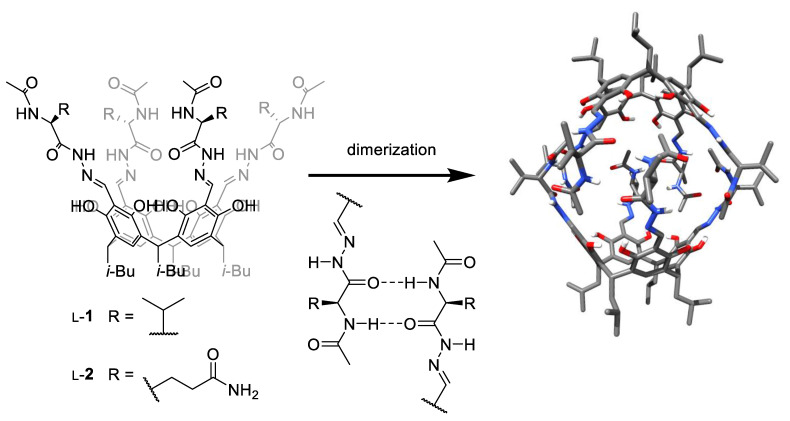
The structures of cavitands used in this study (L-**1** and L-**2**), the dimerization process, and the X-ray structure of dimer (L-**1**)_2_ (from ref. [12]).

We have previously reported the synthesis and self-assembly of various peptide-based capsules, which are formed by hydrogen bonds between the peptide strands, forming β-sheet-like binding motifs [11,12,13,14,15]. Self-assembly of valine derivative L-**1** (Figure 1) in non-polar solvents (chloroform) proceeds quantitatively and leads to a dimeric capsule (L-**1**)_2_ [12]. In this environment, the open/close processes are hindered, and the encapsulation of guests does not proceed due to the high kinetic barrier; therefore, various methods of activation are required to induce encapsulation, e.g., mechanochemical pre-treatment [16]. At the other extreme, the self-assembly of hydrophilic analogs, such as glutamine derivative L-**2,** in a polar environment (water, methanol, DMSO) does not proceed. For effective self-assembly in the polar environment, the presence of a template (fullerene) is required, which, however, blocks future encapsulation of the guests [13,15]. Seeking the conditions under which self-assembly remains efficient but is more dynamic (lower kinetic barriers), we tested the self-assembly of hydrophobic derivative L-**1** and hydrophilic L-**2** in solvent mixtures with moderate polarity, e.g., in the chloroform/methanol mixtures.

When following the self-assembly of L-**1** or L-**2** in the chloroform/methanol by ^1^H NMR, we have encountered numerous interpretational problems. Hydrogen atoms that are involved in binding motifs undergo deuteration/exchange and are not visible. Moreover, the number of signals in ^1^H NMR spectra is reduced by symmetry; therefore, relevant NOE effects cannot be interpreted and the changes in the chemical shift for protons that are non-hydrogen bonded are small. Additionally, DOSY measurements are affected by strong solvation effects. With these observations, we turned our attention to ECD spectroscopy.

**Figure 2:**
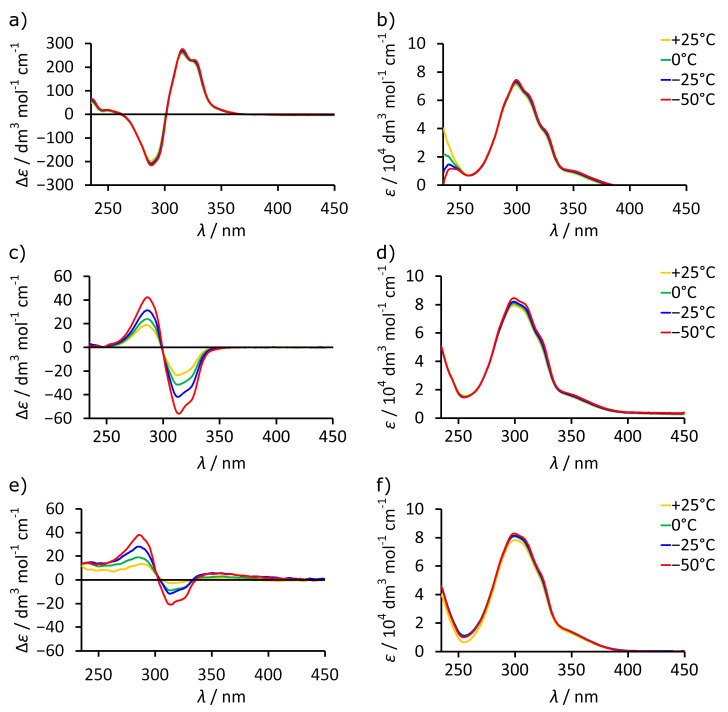
Temperature-dependent ECD and UV spectra in various solvents: (**a**) ECD spectra of L-**1** in CHCl_3_; (**b**) UV spectra of L-**1** in CHCl_3_; (**c**) ECD spectra of L-**1** in MeOH; (**d**) UV spectra of L-**1** in MeOH; (**e**) ECD spectra of L-**2** in MeOH; (**f**) UV spectra of L-**2** in MeOH (all intensities of ECD and UV spectra calculated for the concentration of the cavitand).

It should be noted that all intensities of UV and ECD spectra reported in this paper were calculated for the concentration of cavitand to make the data independent on the dimerization and normalized for the number of chromophores. ECD spectrum of L-**1** in chloroform, in which the dimeric capsule (L-**1**)_2_ is quantitatively formed, exhibits strong ECD bands in the range of 250–350 nm with intensities reaching Δ*ε* ≈ ±250 dm^3^ mol^−1^ cm^−1^ (Figure 2a,b). In contrast, the ECD spectra of L-**1** and L-**2** in methanol at +25 °C show low intensity (Δ*ε* < ±20 dm^3^ mol^−1^ cm^−1^) and the opposite signs to those in chloroform, while the UV spectra remain at the same level (Figure 2c–f). Importantly, upon lowering the temperature from +25 °C to −50 °C, the ECD spectra in chloroform remain unchanged, but in methanol, the intensities of the spectra for both L-**1** and L-**2** increase, while their signs remain opposite to those in chloroform. Thus, the ECD spectra indicate that the dimeric species (L-**1**)_2_ formed in chloroform and monomeric species L-**1** and L-**2** formed in methanol show distinct ECD characteristics in regard to sign, intensity, and temperature-dependent response. Therefore, we expect that these features will be useful for the characterization of the self-assembly process.

**Figure 3:**
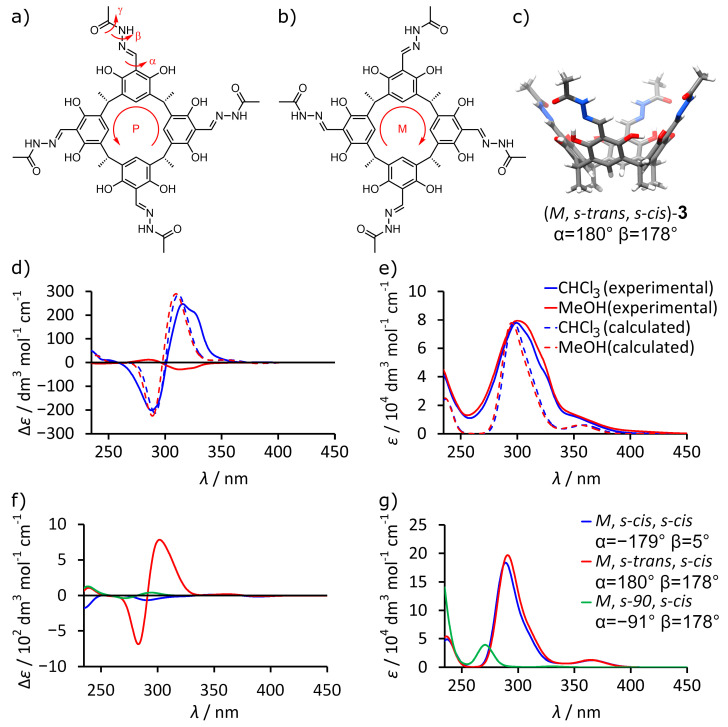
Comparison of experimental spectra of L-**1** and theoretical spectra of **3** in CHCl_3_ and MeOH: (**a**–**c**) models used for estimation of the influence of conformational effects (coordinates available in Supplementary Materials); (**d**) experimental and theoretical ECD and (**e**) UV spectra; (**f**) calculated ECD and (**g**) UV spectra for different conformations of **3**. The intensities of experimental spectra were calculated for the concentration of cavitand L-**1**. Theoretical spectra were calculated using TD DFT B3LYP/6-31G(d,p), starting from the geometry retrieved from the crystal structure; their intensities (ECD and UV) were normalized at intensity of experimental UV spectra.

To reveal the reasons for the observed ECD effects, we analyzed the possible structural factors and performed a series for theoretical calculations (TD DFT) [17]. The hypothesis is that ECD effects originate from the inherently chiral conformation of acylhydrazone/resorcinol chromophores induced by the chirality of the attached peptide arms. Our assumption is based on the observation that the high intensity of the bands in the range of 250–350 nm is unusual, considering that stereogenic centers are positioned at the peptide arms which contain only amide-type chromophores (that do not absorb in this region). The acylhydrazone/resorcinol part, which is bridged by a single bond, can undergo rotation (α, Figure 3a,b), and rotation about α creates an inherently chiral (*P* or *M)* conformation. To check this, we constructed model molecule **3** (Figure 3a–c), which is devoid of the chiral peptide arms, but only exhibits inherently chiral conformation. Model structure **3** has three rotatable, formally single bonds per unit (torsion angles α, β and γ, Figure 3a). Angle α is crucial as it defines *M*/*P* stereochemistry. Angle β defines *s-cis*/*trans* stereochemistry around the hydrazine bond N-N. Angle γ defines stereochemistry of an amide bond, and it was fixed to be *s-cis* (γ = 180°).

In the crystal structure of L-**1,** the conformation of the acylhydrazone/resorcinol part is (*M*, *s-trans*, *s-cis*). The theoretical ECD spectrum of (*M, s-trans, s-cis*)-**3** calculated using TD DFT B3LYP/6-31g(d,p) in chloroform (PCM solvation model) has bands in the range of 250–350 nm that correspond well to the experimental spectrum of (L-**1**)_2_ in chloroform concerning the energies, signs, and intensities (Figure 3d,e). Importantly, the change in the solvent to methanol does not alter the theoretical ECD spectrum significantly, indicating that non-specific solvent effects cannot be responsible for the dramatic reduction in intensity and reversal of signs observed in the experimental ECD spectrum in methanol.

To further analyze the structural changes that influence the ECD spectrum, we calculated theoretical spectra for various hypothetical *C*_4_-symmetric conformations of **3**. The results show that the change in angle β from 180° to 0° (*s-trans* to *s-cis*) significantly lowers the intensity of CD bands (Figure 3f,g). Additionally, changes in α from 180° to 90° greatly reduce the intensity of ECD bands; however, the change in α from 180° to 0° (*M* to *P*) results in reversal of the signs of ECD bands. These theoretical calculations demonstrate that not only signs, but also intensities of the ECD bands are correlated with conformational changes and suggest that any type of conformational lability should result in lowering the intensity of the ECD effects.

**Figure 4:**
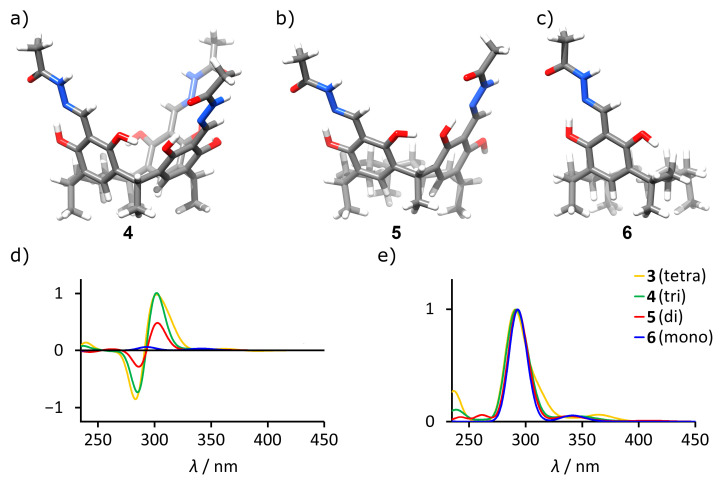
The influence of the number of units on the ECD spectra theoretical calculations (TD DFT B3LYP/6-31G(d,p)). (**a**–**c**) Models used for estimation of the influence of the number of units (coordinates available in Supplementary Materials); (**d**) calculated ECD spectra; (**e**) calculated UV spectra. The intensities of theoretical spectra were normalized at the intensity of UV spectra of (*M, s-trans, s-cis*)-**3**.

Having established the dependence of ECD on the conformation of macrocycle **3**, which possesses four chromophoric acylhydrazone/resorcinol units separated by methylene bridges, we studied the effect of the number of chromophores and their spatial arrangements on possible amplification caused by a macrocyclic structure. Therefore, hypothetical macrocyclic molecules **4**–**6** were constructed having one, two, or three chromophoric units, respectively, while the remaining part was replaced by an aliphatic linker (Figure 4a–c). To account for a different number of chromophores, the obtained ECD spectra were normalized using theoretical UV spectra (Figure 4d,e). The results demonstrate that indeed there is a substantial amplification effect linked to the increase in the number of units and the maximum intensity is reached already for the macrocycle containing three units.

These calculations allow us to conclude that the sign and the intensity of bands in the range of 250–350 nm are correlated not only to the conformation of L-**1,** but also to its self-assembly because self-assembly strengthens the induction of the inherently chiral conformations and restricts conformational lability around α.

**Figure 5:**
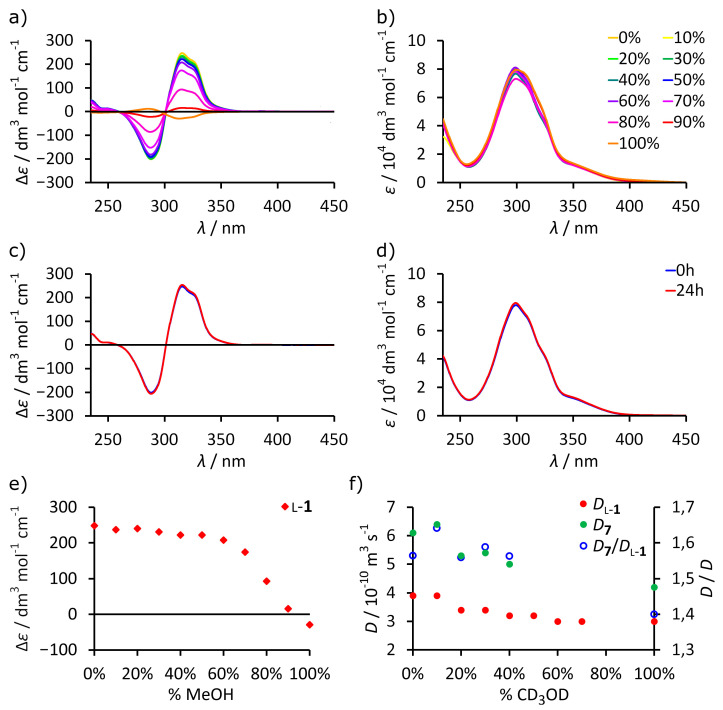
Experimental UV/ECD and DOSY spectra for L-**1** in solvent mixtures at 25 °C: (**a**) ECD spectra in CHCl_3_/MeOH; (**b**) UV spectra in CHCl_3_/MeOH; (**c**) time-dependent ECD spectra in CHCl_3_/MeOH 30%; (**d**) time-dependent UV spectra in CHCl_3_/MeOH 30%; (**e**) changes in ECD intensities dependent on the amount MeOH in CHCl_3_ (all intensities of ECD and UV spectra calculated for the concentration of cavitand); (**f**) changes in diffusion coefficients (*D*) from DOSY spectra dependent on the amount CD_3_OD in CDCl_3_.

**Figure 6:**
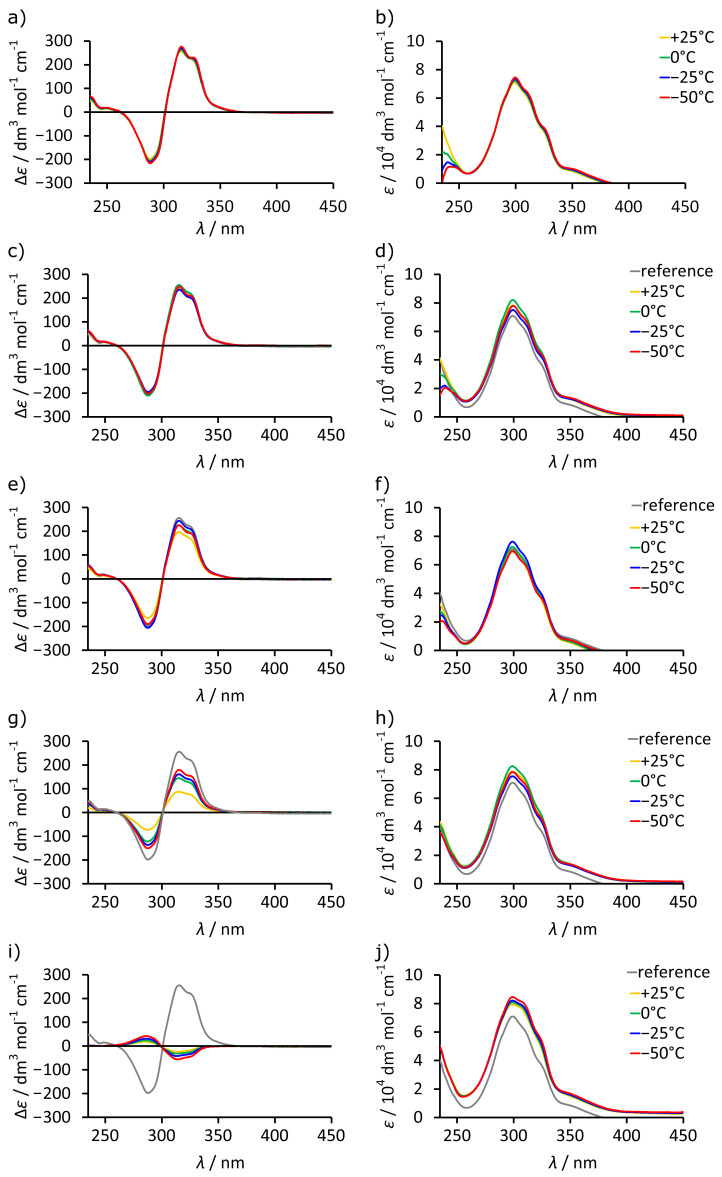
Temperature-dependent ECD and UV spectra of L-**1** in various solvents: (**a**) ECD spectra in CHCl_3_; (**b**) UV spectra in CHCl_3_; (**c**) ECD spectra in CHCl_3_/MeOH 30%; (**d**) UV spectra in CHCl_3_/MeOH 30%; (**e**) ECD spectra in CHCl_3_/MeOH 60%; (**f**) UV spectra in CHCl_3_/MeOH 60%; (**g**) ECD spectra in CHCl_3_/MeOH 80%; (**h**) UV spectra in CHCl_3_/MeOH 80%; (**i**) ECD spectra in MeOH 100%; (**j**) UV spectra in MeOH 100% (all intensities of ECD and UV spectra calculated for the concentration of cavitand). The reference spectrum is the experimental spectrum of L-**1** in CHCl_3_ at 25 °C.

Having established the dependence of the sign and intensity of ECD on the efficiency of self-assembly of L-**1** we utilized this knowledge to study self-assembly in mixed solvents. Upon a gradual increase in the fraction of MeOH in chloroform (from 0 to 100%), the intensity of ECD bands for L-**1** decreases, while the intensity of the UV spectra stays at the same level (Figure 5a,b). However, the decrease is not linear, and the intensity remains high up to 60% of MeOH (Figure 5e). Control measurements excluded kinetic effects (Figure 5c,d). It should also be noted that reversal of the sign of ECD spectra is not observed until 90% of MeOH. The temperature-dependent ECD spectra were recorded for the samples containing 0%, 30%, 60%, 80%, and 100% of MeOH (Figure 6). In all cases, the intensity of the bands gradually increases upon lowering the temperature. For up to 60% MeOH, the intensity reached at −50 °C (which is the experimental limit) is almost as high as in pure chloroform (Δ*ε_max_*). For 80% MeOH, the intensity changes from 0.34 Δ*ε_max_* at +25 °C to 0.70 Δ*ε_max_* at −50 °C. It is important to note that in MeOH (100%), the sign of the changes for L-**1** is the opposite and the increase in the intensity of the bands was much less pronounced. This leads us to the conclusion that self-assembly of L-**1** into dimeric (L-**1**)_2_ is very efficient in up to 60% of methanol in chloroform, and it increases at lower temperatures. For 80% MeOH, self-assembly is detectable but less efficient. However, in 100% MeOH, there is no detectable evidence for self-assembly; instead, L-**1** shows the changes that are typical for non-specific temperature-dependent lowering of conformational freedom. Such an abrupt change in intensity as is observed between 80–100% of MeOH cannot be explained by a simple change in polarity and suggests that chloroform plays a special role in the self-assembly process. We are not able to rationalize it at this moment, but we think that chloroform molecules may be encapsulated and may act as templates.

With the solid conclusions that were drawn using ECD, we re-investigated DOSY results. L-**1** has limited solubility in MeOH at the concentration that is required for NMR, which precluded obtaining some data points. For the same reason, the concentrations of L-**1** were not constant. To make the data more reliable, we used an internal standard—resorcinarene tetraaldehyde **7**. Tetraaldehyde **7** is a substrate in the reaction leading to L-**1** and it does not self-assemble; therefore, it is a good internal standard. Diffusion coefficients (*D*) that are measured by DOSY depend on the viscosity, *η*, of the solvents according to the Stokes–Einstein equation (*D*~1/*η*). However, methanol and chloroform have almost the same *η*, so viscosity changes should not affect *D* values in the solvent mixtures. Despite this, we have found that *D* values for reference compound **7** undergo substantial changes with an increasing fraction of methanol, suggesting non-linear solvation effects (Figure 5f). To balance these effects for samples containing L-**1,** we calculated *D*(**7**)/*D*(L-**1**) for each data point, which should reflect the relative ratio between radii of the species *r_H_*(L-**1**)/*r_H_*(**7**). The data indicate that for CDCl_3_/CD_3_OD (0–40%), *r_H_*(L-**1**)/*r_H_*(**7**) > 1.55, while for MeOD, experimental *r_H_*(L-**1**)/*r_H_*(**7**) = 1.4, which roughly follows the values obtained for models *r_H_*(L-**1**)_2_/*r_H_*(**7**) = 2.0 *r_H_*(L-**1**)/*r_H_*(**7**) = 1.76. However, it should be noted that without ECD data, interpretation of DOSY spectra, especially from a single measurement, would be ambiguous.

## 3. Materials and Methods

Compounds L-**1**, L-**2**, and **7** were obtained according to the literature procedures [12,13,18].

ECD spectra were recorded on an ECD Jasco J-715 spectropolarimeter. All measurements were made in a 0.2 cm cuvette. Concentrations of all samples were 7 × 10^−5^ mol dm^−3^.

All calculations were performed within the density functional theory (DFT) using Gaussian 09 program suite [17]. Excited electronic states were determined at the B3LYP/6-31G(d,p) level by means of the time-dependent DFT (TD DFT) approach (100 excited states). The ECD and UV spectra were simulated by overlapping Gaussian functions for each transition where the width of the band at 1/e height is fixed at 0.16 eV.

NMR DOSY experiments were performed on a Varian VNMRS-600 spectrometer (Palo Alto, CA, USA) equipped with a 5 mm PFG AutoXID (^1^H/X^15^N-^31^P) probe at 298 K. DOSY measurements were run using the DPFGDSTE pulse sequence (with convection compensation) for measurements in CDCl_3_ and other mixtures of CDCl_3_/CD_3_OD. The gradient strengths were incremented as a square dependence in the range from 6 to 55 G cm^−1^. Sixteen transients were recorded for each increment, with a 3.2 s acquisition time and 1 s relaxation delay (for an overall experiment time of 20 min). The duration of magnetic field gradients (*δ*) was 2 ms, whereas the diffusion delay (Δ) was chosen as 80 ms for CDCl_3_/CD_3_OD and mixtures of them. Other parameters include the following: a sweep width of 12,000 Hz, 32 K data points. The data were processed using Varian DOSY software [19].

## 4. Conclusions

In conclusion, we have shown that electronic circular dichroism spectroscopy can be used for monitoring self-assembly for confrontationally labile chiral cavitands, and all parameters of the spectra (sign, intensity, and temperature response) are sensitive probes of this process. Moreover, by cumulative analysis of experimental data and theoretical calculations, it has been found that the differences between assembled and non-assembled species originate from the induction of an inherently chiral conformation and restriction of conformational freedom in the self-assembled dimer (L-**1**)_2_, as compared to the monomeric species L-**1**. Therefore, restriction of intramolecular motion (RIM) is the mechanism of amplification of ECD signals during the self-assembly of discrete species.

## Data Availability

The date are available on request from the corresponding author.

## Supplementary Materials

The following are available online, atom coordinates of models **3**–**6**.

## Sample Availability

Samples of the compounds L-**1** and L-**2** are available from the authors.
