# Supplementary material for: Amplification of Electronic Circular Dichroism—A Tool to Follow Self-Assembly of Chiral Molecular Capsules
Source: Molecules. 2021 Nov 24;26(23):7100. doi: 10.3390/molecules26237100 (PMC8658961; doi:10.3390/molecules26237100)
Supplement: Supplementary file 1 [file molecules-26-07100-s001.zip › molecules-1470968-supplementary.pdf]

SUPPLEMENTARY INFORMATION

Amplification of electronic circular dichroism– a tool to follow self-assembly of chiral molecular capsules

Marek P. Szymański, Marcin Grajda and Agnieszka Szumna*

Atomic Cartesian coordinates for all calculated geometries:

(*M, s-cis, s-cis*)-**3**   α = -179 ° β = 5 °

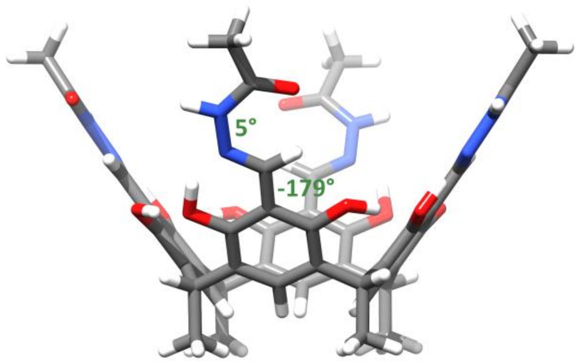

C	0.01152600	-6.77403700	3.28712400
C	1.28670700	-3.00164300	-2.04176000
C	0.04828400	-2.62490400	-2.57191700
H	0.04364200	-1.96674600	-3.43420400
C	-1.18245200	-3.05684700	-2.07303000
C	-1.15618400	-3.91791900	-0.96936700
C	0.06207000	-4.31873000	-0.36903200
C	1.28225400	-3.84163000	-0.91360400
C	0.10920800	-5.16406200	0.81318800
H	1.07562000	-5.38093700	1.25113500
C	-3.00280700	-1.28702700	-2.04399900
C	-2.62515800	-0.04863200	-2.57356200
H	-1.96511300	-0.04399900	-3.43440900
C	-3.05826600	1.18211100	-2.07571900
C	-3.92163300	1.15587100	-0.97386100
C	-4.32351600	-0.06231600	-0.37420300
C	-3.84506300	-1.28252600	-0.91753100
C	-5.17106400	-0.10913500	0.80642600
H	-5.38818800	-1.07535400	1.24470300
C	-1.28729400	3.00098100	-2.04433500
C	-0.04867800	2.62438500	-2.57414700
H	-0.04372400	1.96647400	-3.43662600
C	1.18187400	3.05602700	-2.07454800
C	1.15515400	3.91661400	-0.97050500
C	-0.06331500	4.31716700	-0.37047200
C	-1.28329000	3.84032700	-0.91573200
C	-0.11077600	5.16169900	0.81228500
H	-1.07731200	5.37847700	1.25001100
C	3.00251100	1.28664600	-2.04423100
C	2.62453700	0.04785000	-2.57262200
H	1.96428800	0.04254100	-3.43331900
C	3.05772800	-1.18249700	-2.07387000

C	3.92144900	-1.15545000	-0.97229500
C	4.32365400	0.06325900	-0.37376500
C	3.84519100	1.28301100	-0.91809900
C	5.17196500	0.11152500	0.80631600
H	5.38859300	1.07828200	1.24364600
N	-1.01748400	-6.40180800	2.45025100
N	-0.99777200	-5.61959400	1.31841800
N	-5.62837600	0.99797000	1.30978400
N	0.99609500	5.61702200	1.31793400
N	5.63053100	-0.99470800	1.31040900
O	2.41699700	-4.27107600	-0.30690400
H	3.14783700	-3.62455100	-0.41149700
O	-2.34493700	-4.36070200	-0.46402900
H	-2.13531100	-4.94329000	0.32425200
O	-4.27532000	-2.41728200	-0.31142500
H	-3.62770000	-3.14745300	-0.41390200
O	-4.36557900	2.34457400	-0.46940300
H	-4.95021600	2.13450300	0.31721300
O	-2.41834200	4.26907900	-0.30911300
H	-3.14898000	3.62245300	-0.41461400
O	2.34367600	4.35898400	-0.46421400
H	2.13356600	4.94145900	0.32400200
O	4.27626600	2.41832400	-0.31356800
H	3.62928000	3.14892600	-0.41717000
O	4.36519400	-2.34395900	-0.46718600
H	4.94925100	-2.13394000	0.31985500
N	6.41578000	-1.01294600	2.44015100
C	6.78788700	0.01673900	3.27624100
N	1.01551600	6.39870600	2.45014000
C	-0.01360800	6.76998600	3.28729000
N	-6.41270600	1.01833800	2.44022500
C	-6.78426200	-0.00959100	3.27881100
C	2.52068800	2.60434000	-2.66307100
H	3.25518600	3.36689900	-2.38827500
C	2.60483300	-2.52152400	-2.66098400
H	3.36678700	-3.25636000	-2.38541400
C	-2.52105700	-2.60514100	-2.66202800
H	-3.25574600	-3.36745400	-2.38704600
C	-2.60521600	2.52065800	-2.66376400
H	-3.36719300	3.25574500	-2.38891800
C	2.57534900	-2.51706400	-4.20083200
H	2.30584300	-3.50858100	-4.57610800
H	3.56178200	-2.25344700	-4.59343100
H	1.85800900	-1.80370300	-4.61665400
C	-2.51474700	-2.57624200	-4.20187800
H	-3.50605200	-2.30775900	-4.57844600
H	-2.24976600	-3.56257100	-4.59382500
H	-1.80156100	-1.85837200	-4.61708200

C	-2.57518100	2.51506800	-4.20359300
H	-2.30564300	3.50633400	-4.57951000
H	-3.56144400	2.25105200	-4.59635200
H	-1.85760800	1.80148200	-4.61863000
C	2.51466600	2.57464600	-4.20290200
H	3.50613400	2.30629900	-4.57913500
H	2.24943700	3.56069200	-4.59539500
H	1.80179200	1.85633300	-4.61788200
C	-0.43741000	-7.60770400	4.47811500
H	-1.50665100	-7.83515400	4.49055900
H	-0.17940100	-7.07125000	5.39527300
H	0.12489700	-8.54485900	4.47644500
O	1.18299500	-6.47261000	3.10687300
O	6.48435300	1.18771600	3.09632100
O	-1.18495500	6.46813700	3.10694800
O	-6.48149500	-1.18108300	3.10092600
C	-7.61913200	0.44068400	4.46846000
H	-8.55408000	-0.12524300	4.46942400
H	-7.85061300	1.50909600	4.47685800
H	-7.08126700	0.18836000	5.38639800
C	0.43525100	7.60201500	4.47945500
H	-0.12919700	8.53785600	4.48062300
H	1.50400200	7.83182500	4.49076100
H	0.17991000	7.06300200	5.39588200
C	7.62512000	-0.43063200	4.46532500
H	7.08895400	-0.17640200	5.38372600
H	8.55987100	0.13562100	4.46315800
H	7.85704100	-1.49893500	4.47569800
H	-1.95605400	-6.67520500	2.69507300
H	-6.68687600	1.95704600	2.68371800
H	1.95402600	6.67202200	2.69529300
H	6.69125100	-1.95103700	2.68447800

(*M, s-trans, s-cis*)-**3** α = -180 ° β = 178 °

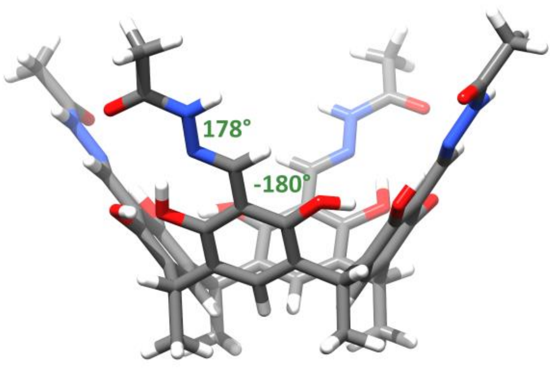

C	-2.40778600	-3.62630100	2.53870300
C	-2.85042500	-3.12414100	1.15269700

H	-2.06181800	-2.51077000	0.70653800
C	-4.14764100	-2.30628000	1.23181900
C	-3.65425100	-0.40966900	-0.41194200
C	-2.60936000	-0.49322900	-1.34310000
H	-2.46763600	-1.43228700	-1.86518800
C	-1.74057300	0.55761000	-1.64291200
C	-1.94090700	1.77262400	-0.96977300
C	-2.98460500	1.92308000	-0.02201900
C	-3.82032200	0.81646400	0.24611900
C	-3.20719000	3.17299300	0.68079000
H	-4.03219000	3.21355800	1.39653300
C	0.68945300	-0.10739300	-1.93549700
C	0.92362300	-1.48226100	-1.76910700
H	0.17993500	-2.17378300	-2.15059300
C	2.05980900	-2.01872300	-1.16727800
C	3.03247600	-1.11269200	-0.69439300
C	2.84317300	0.28689200	-0.83149600
C	1.66080800	0.77466100	-1.44465700
C	3.81976300	1.23845100	-0.34072900
H	3.58485000	2.29938400	-0.46197600
C	1.89536400	-3.99583000	0.43328400
C	0.38880700	-4.01728600	0.73062400
H	-0.04214300	-3.04584900	0.46622200
C	0.09894300	-4.33838900	2.20765800
N	-2.44777800	4.19276900	0.45923400
N	4.91630200	0.84324400	0.21610300
O	-4.79578600	1.02332600	1.18661000
H	-5.32840800	0.22292800	1.27332700
O	-1.10684600	2.80938000	-1.24957200
H	-1.36763700	3.60128600	-0.70087700
O	1.56075400	2.13057100	-1.55292900
H	0.62504700	2.41384400	-1.52715200
O	4.13186100	-1.62273700	-0.10702000
H	4.73127900	-0.88178400	0.16365300
N	5.79289000	1.78692700	0.66094100
C	6.97506000	1.41257600	1.27091300
N	-2.67499000	5.35233000	1.12969400
C	-1.85455900	6.44818100	0.91273800
C	2.29761000	-3.51347600	-0.98789100
H	3.38255600	-3.65293900	-1.04573800
C	-1.34062500	-4.74701400	2.60595700
H	-1.27204800	-4.99465700	3.67569700
C	-4.57021700	-1.58568800	-0.08133900
H	-5.57658600	-1.17183600	0.10604200
C	-0.57618900	0.41228600	-2.62776300
H	-0.34915700	1.41876600	-2.99034000
C	-1.80187100	-6.03459000	1.90226400
H	-2.77520000	-6.36263000	2.28462800

H	-1.08720800	-6.84705000	2.07315600
H	-1.90011500	-5.91198200	0.81925100
C	-4.76688700	-2.56694800	-1.24761700
H	-5.12912000	-2.04816600	-2.14000300
H	-5.50243800	-3.33044100	-0.97470100
H	-3.84399200	-3.08805700	-1.51468800
C	-0.95155700	-0.40200200	-3.87968600
H	-0.11289300	-0.41331500	-4.58135400
H	-1.81542300	0.04726100	-4.37914100
H	-1.20374700	-1.44287700	-3.65843100
C	1.67612400	-4.37649000	-2.09717900
H	1.95105600	-5.42723200	-1.95416500
H	2.03472300	-4.06525000	-3.08339800
H	0.58244700	-4.33079400	-2.11327000
O	7.29201000	0.25082100	1.45049800
O	-0.92018400	6.42385500	0.13414200
C	-2.22890200	7.67188300	1.72974900
H	-1.36699100	7.96443100	2.33497200
H	-3.09038300	7.52360200	2.38677800
H	-2.44321000	8.49638700	1.04474400
C	7.84574100	2.58344500	1.69795900
H	8.02638100	2.50965000	2.77351700
H	7.41690100	3.56491100	1.47654600
H	8.81373100	2.49909300	1.19687700
H	-3.44294200	5.39837800	1.79217200
H	5.56529700	2.76792100	0.53029500
H	-0.09707300	-4.75962500	0.08614000
H	0.40316600	-3.47916200	2.82111500
H	0.75802600	-5.16462600	2.51106100
H	2.29925700	-5.00769600	0.58055000
H	2.40507000	-3.35574900	1.16312500
H	-3.00284000	-3.97552500	0.48015000
H	-3.29837500	-4.00232100	3.06377000
H	-2.05547000	-2.76848600	3.12799300
H	-4.04314200	-1.55385500	2.02599900
H	-4.96831000	-2.96773500	1.54314200

(*M, s-90, s-cis*)-**3** α = -91 ° β = 178 °

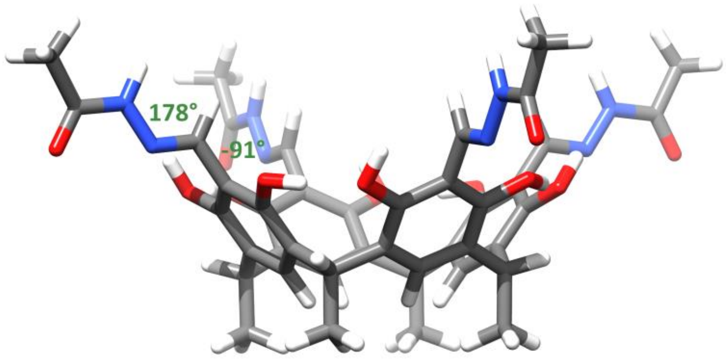

C	-9.81682892	2.28439475	0.68760369
C	-3.42453137	0.75697827	2.49505085
C	-3.51083496	0.09877073	3.72694998
H	-2.61925791	0.05788419	4.34368222
C	-4.67457361	-0.49307801	4.22225464
C	-5.82138700	-0.42358770	3.42023449
C	-5.79236740	0.21488638	2.15620074
C	-4.58146925	0.79317747	1.69873529
C	-6.96274376	0.26535698	1.30213981
H	-7.10297535	-0.55575546	0.59505987
C	-4.31761241	-2.69247370	5.43773335
C	-2.98205844	-3.09780781	5.53917106
H	-2.23095324	-2.33259602	5.70416927
C	-2.55503884	-4.42494927	5.46001116
C	-3.53674523	-5.40399694	5.25753507
C	-4.90341017	-5.05422680	5.13075971
C	-5.28080868	-3.69005839	5.21167418
C	-5.92524932	-6.05367186	4.88858147
H	-6.15691899	-6.29158156	3.84762668
C	-0.38251120	-4.77233323	4.19444381
C	0.21600249	-3.59903078	3.72172014
H	0.16299726	-2.71477905	4.34799823
C	0.89274585	-3.49693604	2.50453081
C	0.96280029	-4.64932061	1.71057801
C	0.36238886	-5.86246566	2.12756199
C	-0.31897701	-5.90821642	3.36996200
C	0.39934337	-7.05299301	1.30116091
H	-0.41753066	-7.19465885	0.58946834
C	0.51042138	-1.32299249	1.25149267
C	-0.31277630	-0.40236370	1.90958446
H	-0.22507592	-0.32395223	2.98805264
C	-1.22690515	0.43487938	1.26667746
C	-1.32211906	0.33083565	-0.12738090
C	-0.52702778	-0.59377541	-0.84785746
C	0.37985291	-1.42542352	-0.14367512
C	-0.63871930	-0.73437088	-2.28641372
H	-1.36427534	-1.45863631	-2.66439142

N	-8.89283356	1.26041504	0.58255951
N	-7.79897318	1.24543804	1.39199143
N	-6.52233012	-6.63426520	5.87564876
N	1.37000778	-7.89781894	1.41053656
N	0.09272254	-0.01891099	-3.07443954
O	-4.63325619	1.39736967	0.48367562
H	-3.76004735	1.38174329	0.03407876
O	-6.96860868	-0.99823913	3.88265662
H	-7.69273995	-0.87383941	3.20369917
O	-6.60891988	-3.43295073	5.09283377
H	-6.77293910	-2.53692019	4.72530388
O	-3.14200904	-6.70678620	5.17331535
H	-3.94625304	-7.28252653	5.02323557
O	-0.85875945	-7.10762668	3.70782743
H	-1.64114316	-7.00089074	4.29208723
O	1.61584708	-4.57180131	0.51570113
H	1.57691990	-5.46356595	0.06401180
O	1.11587262	-2.27803365	-0.90224912
H	1.37105659	-3.08219019	-0.39916679
O	-2.21120072	1.13646239	-0.77600613
H	-2.16992007	0.94479037	-1.75700507
N	-0.04140460	-0.16703318	-4.42054492
C	0.71021730	0.61341352	-5.28034163
N	1.38139956	-9.00172156	0.61480602
C	2.42958970	-9.90075834	0.69439028
N	-7.47113496	-7.57474562	5.61641287
C	-8.09772219	-8.23100024	6.66044924
C	1.51923858	-2.18931489	2.01391831
H	2.29841761	-2.46716103	1.29836970
C	-2.12356311	1.41701353	2.02459312
H	-2.40680718	2.19475554	1.30959719
C	-4.72403125	-1.22030421	5.56824326
H	-5.76975988	-1.21052414	5.88868773
C	-1.08120269	-4.82657140	5.55769285
H	-1.06445626	-5.87235694	5.87766577
C	-1.38157756	2.14961181	3.15787856
H	-2.04277368	2.88279231	3.62879693
H	-0.51045350	2.67549891	2.75646163
H	-1.02382233	1.47902673	3.94445271
C	-3.94730151	-0.48184451	6.67410605
H	-4.06217099	-1.00416894	7.62829695
H	-4.33286073	0.53558136	6.78696715
H	-2.87539660	-0.40372021	6.47091648
C	-0.32222811	-4.04945692	6.64930415
H	0.70684435	-4.41269071	6.72392176
H	-0.81092836	-4.19040481	7.61773411
H	-0.27628714	-2.97302100	6.46077137
C	2.24353665	-1.41817937	3.13298497

H	2.72622223	-0.52578362	2.72415328
H	3.01170757	-2.05084743	3.58706819
H	1.57525640	-1.09049263	3.93448077
C	-10.97188545	2.17830414	-0.29288319
H	-10.95028133	1.27724542	-0.91210371
H	-10.95808803	3.05553418	-0.94552500
H	-11.90917964	2.20208020	0.26849474
O	-9.70700033	3.19104795	1.49308781
O	1.49800160	1.45498246	-4.88789588
O	3.37233073	-9.74862809	1.45001881
O	-7.83200532	-8.01465615	7.82898052
C	-9.14660195	-9.23659168	6.21843006
H	-8.90925971	-10.20621127	6.66308805
H	-9.22549511	-9.35172821	5.13384545
H	-10.11791377	-8.92437192	6.61183757
C	2.29475008	-11.09221422	-0.23780836
H	3.19482617	-11.15623541	-0.85423088
H	1.41818125	-11.05148149	-0.89032204
H	2.24436448	-12.00342496	0.36456572
C	0.46923420	0.32159093	-6.75120519
H	1.40469494	-0.02897801	-7.19589331
H	0.19790962	1.25435738	-7.25172751
H	-0.31045819	-0.42230979	-6.93692601
H	-9.01769004	0.51050048	-0.09018749
H	-7.70439345	-7.78561268	4.65117823
H	0.61496247	-9.14838954	-0.03451539
H	-0.70007842	-0.85226317	-4.77714623

4

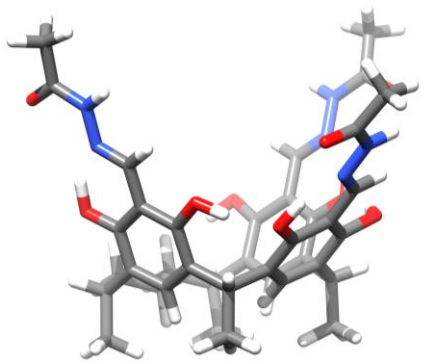

C	5.17055200	-1.92581500	-4.14774800
C	0.05469900	-4.29102000	0.34762300
C	0.64638200	-3.55426600	1.38440900
H	0.19535400	-3.61159700	2.36776100
C	1.78600400	-2.76392800	1.23212900
C	2.38242700	-2.72585800	-0.03848400
C	1.82652000	-3.44011700	-1.12877800

C	0.65677400	-4.20447700	-0.91390900
C	2.42034900	-3.39893300	-2.45184300
H	1.92817300	-3.96217500	-3.24854000
C	1.96824600	-0.45791900	2.26044900
C	0.68992000	-0.03730500	2.64601400
H	-0.01695800	-0.78912800	2.98539000
C	0.26488000	1.29148700	2.61778700
C	1.17355600	2.25102700	2.14964300
C	2.47226900	1.88030700	1.72232200
C	2.85220900	0.51786400	1.77598100
C	3.40417200	2.85191900	1.18307100
H	4.37245400	2.47890800	0.84024000
C	-2.17135300	1.33323900	1.95032900
C	-2.99144200	0.20004500	2.05260400
H	-2.91446300	-0.39844000	2.95318100
C	-3.91291800	-0.19536600	1.08334800
C	-4.01706200	0.60116700	-0.07369600
C	-3.22362400	1.76908800	-0.22229600
C	-2.30014800	2.12058900	0.79599000
C	-3.33115300	2.61532100	-1.39325800
H	-2.69218900	3.50181600	-1.42920300
C	-4.25480800	-2.64248200	0.42708800
C	-2.93136400	-3.23387600	0.93326900
H	-2.14733000	-2.46847700	0.90289200
C	-2.49938900	-4.45061300	0.10414400
N	4.03297000	-2.67992100	-3.91020300
N	3.49229400	-2.71190500	-2.66407100
N	3.08157900	4.09983900	1.10959100
N	-4.15050200	2.33103800	-2.35104700
O	0.16895500	-4.86043400	-2.01296300
H	-0.64652100	-5.31936000	-1.77616200
O	3.51301600	-1.98984900	-0.20080800
H	3.81400300	-2.05476900	-1.15170200
O	4.11256800	0.21594100	1.34759800
H	4.07073600	-0.61894400	0.83827500
O	0.75149100	3.54456500	2.07680600
H	1.48959500	4.10768500	1.71122900
O	-1.59084300	3.25827200	0.57082900
H	-0.85262500	3.39165700	1.19717700
O	-4.89523100	0.21807600	-1.02193500
H	-4.87816100	0.87255800	-1.76566500
N	-4.21008700	3.17237900	-3.42209400
C	-5.05805200	2.90211400	-4.47833900
N	3.97379100	4.98379200	0.58679500
C	3.63819700	6.32161800	0.47409100
C	-4.80501400	-1.42346100	1.21695600
H	-5.74713900	-1.16495200	0.72044900
C	-1.18678700	-5.15671600	0.55159700

H	-1.07554000	-6.03578800	-0.10599300
C	2.38433900	-1.92988300	2.36554500
H	3.47046700	-1.96233900	2.23895600
C	-1.15211600	1.70172600	3.03137200
H	-1.14973900	2.79257500	3.11879100
C	-1.29898300	-5.74538600	1.96765300
H	-0.39423600	-6.29927600	2.23497100
H	-2.14847900	-6.43380600	2.01978900
H	-1.45844700	-4.97830600	2.72985700
C	2.10881000	-2.50504900	3.76667600
H	2.65440900	-1.92601500	4.51672100
H	2.43874100	-3.54687900	3.82773700
H	1.05046900	-2.47225600	4.04112700
C	-1.51169700	1.17974200	4.43433000
H	-2.52154000	1.49423600	4.71454300
H	-0.80829000	1.58104100	5.16943800
H	-1.46951300	0.08896300	4.50767600
C	-5.15210900	-1.78036800	2.67082400
H	-5.87009600	-2.60745900	2.69372400
H	-5.60406600	-0.92728700	3.18628300
H	-4.28147500	-2.09426500	3.25561400
C	5.66237000	-1.98197500	-5.58277800
H	5.05382000	-2.61035900	-6.23887800
H	6.68910500	-2.35706300	-5.58483700
H	5.68395500	-0.96569900	-5.98479600
O	5.72211200	-1.27904300	-3.27669300
O	-5.77236500	1.91676600	-4.52331800
O	2.55849300	6.76212600	0.82320800
C	4.73601700	7.18625300	-0.12122900
H	4.34665100	7.67819300	-1.01637800
H	5.64418900	6.63686200	-0.38441100
H	4.99118000	7.96902800	0.59788200
C	-5.02121900	3.94724700	-5.58198500
H	-4.76596200	3.45243900	-6.52262900
H	-4.31267800	4.76157600	-5.40538500
H	-6.02329800	4.36943900	-5.69586800
H	3.58400800	-3.19916300	-4.65816800
H	4.87761900	4.64423800	0.27296300
H	-3.63111800	4.00656800	-3.42274800
H	-3.04084200	-3.53047000	1.98334000
H	-2.39688300	-4.13652000	-0.94432500
H	-3.30425000	-5.19887800	0.11910600
H	-5.02343300	-3.42884200	0.44185000
H	-4.14226300	-2.34282700	-0.62128200

5

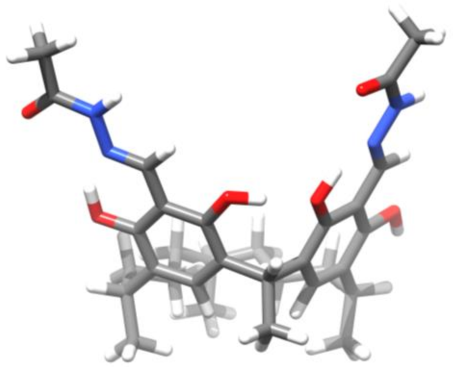

C	-2.40778600	-3.62630100	2.53870300
C	-2.85042500	-3.12414100	1.15269700
H	-2.06181800	-2.51077000	0.70653800
C	-4.14764100	-2.30628000	1.23181900
C	-3.65425100	-0.40966900	-0.41194200
C	-2.60936000	-0.49322900	-1.34310000
H	-2.46763600	-1.43228700	-1.86518800
C	-1.74057300	0.55761000	-1.64291200
C	-1.94090700	1.77262400	-0.96977300
C	-2.98460500	1.92308000	-0.02201900
C	-3.82032200	0.81646400	0.24611900
C	-3.20719000	3.17299300	0.68079000
H	-4.03219000	3.21355800	1.39653300
C	0.68945300	-0.10739300	-1.93549700
C	0.92362300	-1.48226100	-1.76910700
H	0.17993500	-2.17378300	-2.15059300
C	2.05980900	-2.01872300	-1.16727800
C	3.03247600	-1.11269200	-0.69439300
C	2.84317300	0.28689200	-0.83149600
C	1.66080800	0.77466100	-1.44465700
C	3.81976300	1.23845100	-0.34072900
H	3.58485000	2.29938400	-0.46197600
C	1.89536400	-3.99583000	0.43328400
C	0.38880700	-4.01728600	0.73062400
H	-0.04214300	-3.04584900	0.46622200
C	0.09894300	-4.33838900	2.20765800
N	-2.44777800	4.19276900	0.45923400
N	4.91630200	0.84324400	0.21610300
O	-4.79578600	1.02332600	1.18661000
H	-5.32840800	0.22292800	1.27332700
O	-1.10684600	2.80938000	-1.24957200
H	-1.36763700	3.60128600	-0.70087700
O	1.56075400	2.13057100	-1.55292900
H	0.62504700	2.41384400	-1.52715200
O	4.13186100	-1.62273700	-0.10702000
H	4.73127900	-0.88178400	0.16365300
N	5.79289000	1.78692700	0.66094100

C	6.97506000	1.41257600	1.27091300
N	-2.67499000	5.35233000	1.12969400
C	-1.85455900	6.44818100	0.91273800
C	2.29761000	-3.51347600	-0.98789100
H	3.38255600	-3.65293900	-1.04573800
C	-1.34062500	-4.74701400	2.60595700
H	-1.27204800	-4.99465700	3.67569700
C	-4.57021700	-1.58568800	-0.08133900
H	-5.57658600	-1.17183600	0.10604200
C	-0.57618900	0.41228600	-2.62776300
H	-0.34915700	1.41876600	-2.99034000
C	-1.80187100	-6.03459000	1.90226400
H	-2.77520000	-6.36263000	2.28462800
H	-1.08720800	-6.84705000	2.07315600
H	-1.90011500	-5.91198200	0.81925100
C	-4.76688700	-2.56694800	-1.24761700
H	-5.12912000	-2.04816600	-2.14000300
H	-5.50243800	-3.33044100	-0.97470100
H	-3.84399200	-3.08805700	-1.51468800
C	-0.95155700	-0.40200200	-3.87968600
H	-0.11289300	-0.41331500	-4.58135400
H	-1.81542300	0.04726100	-4.37914100
H	-1.20374700	-1.44287700	-3.65843100
C	1.67612400	-4.37649000	-2.09717900
H	1.95105600	-5.42723200	-1.95416500
H	2.03472300	-4.06525000	-3.08339800
H	0.58244700	-4.33079400	-2.11327000
O	7.29201000	0.25082100	1.45049800
O	-0.92018400	6.42385500	0.13414200
C	-2.22890200	7.67188300	1.72974900
H	-1.36699100	7.96443100	2.33497200
H	-3.09038300	7.52360200	2.38677800
H	-2.44321000	8.49638700	1.04474400
C	7.84574100	2.58344500	1.69795900
H	8.02638100	2.50965000	2.77351700
H	7.41690100	3.56491100	1.47654600
H	8.81373100	2.49909300	1.19687700
H	-3.44294200	5.39837800	1.79217200
H	5.56529700	2.76792100	0.53029500
H	-0.09707300	-4.75962500	0.08614000
H	0.40316600	-3.47916200	2.82111500
H	0.75802600	-5.16462600	2.51106100
H	2.29925700	-5.00769600	0.58055000
H	2.40507000	-3.35574900	1.16312500
H	-3.00284000	-3.97552500	0.48015000
H	-3.29837500	-4.00232100	3.06377000
H	-2.05547000	-2.76848600	3.12799300
H	-4.04314200	-1.55385500	2.02599900

H	-4.96831000	-2.96773500	1.54314200

6

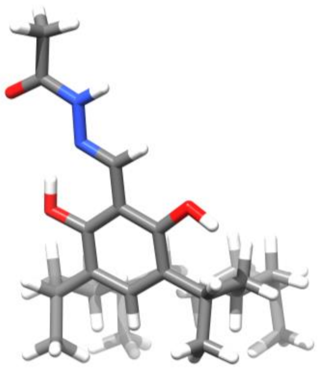

C	3.76273200	0.85608400	1.36648400
H	4.68757500	0.92813800	0.78279600
C	2.52977200	-2.24530500	0.26627400
H	1.99922200	-1.28929500	0.31708000
C	-0.22889900	-1.89312100	-1.28855200
C	0.03809600	-0.64074500	-1.86682600
H	0.94117000	-0.53621200	-2.45590700
C	-0.78834300	0.47413900	-1.74117600
C	-1.97174400	0.32775600	-0.98751400
C	-2.30055600	-0.91904000	-0.39135700
C	-1.41386300	-2.00839400	-0.55401300
C	-3.52112900	-1.10411000	0.36945500
H	-3.70875700	-2.09648600	0.78909900
C	1.41561800	2.49644100	-0.68842600
H	1.33781100	1.51188200	-0.21457900
N	-4.35164400	-0.13036800	0.53994300
O	-1.79251800	-3.18048500	0.05410300
H	-1.17235700	-3.87795900	-0.19165100
O	-2.77315800	1.40162100	-0.86454900
H	-3.56894200	1.15898600	-0.32745600
N	-5.48400400	-0.35616400	1.25965600
C	-6.39736300	0.66411300	1.46234300
C	-0.47598200	1.82603100	-2.37298300
H	-1.43867300	2.23333300	-2.70387700
C	3.24276300	3.43250100	0.99070100
H	3.32195000	4.32386600	1.63044800
C	4.36269200	-1.63135700	2.07866600
H	4.71001500	-2.09165000	3.01543100
C	0.71510700	-3.08613900	-1.42415400
H	0.09048800	-3.98485900	-1.57444200
C	4.37761500	3.54357100	-0.04010800
H	5.35648400	3.55657700	0.45270600
H	4.28605800	4.46860700	-0.62007000

H	4.37993000	2.71064600	-0.74988400
C	5.56236700	-1.65850600	1.11835000
H	5.91377100	-2.68464500	0.96258000
H	6.39991700	-1.07892300	1.52233800
H	5.32104000	-1.24067700	0.13624200
C	1.61676500	-3.02721000	-2.66723500
H	1.02443000	-2.90036500	-3.57811200
H	2.18868900	-3.95615900	-2.75899400
H	2.33753900	-2.20621600	-2.62046800
C	0.42287400	1.73329200	-3.61589100
H	0.52354000	2.72137900	-4.07696900
H	-0.00176900	1.05469500	-4.36265300
H	1.43387700	1.38268200	-3.38472500
O	-6.24250500	1.78761300	1.02181900
C	-7.60915100	0.25247900	2.28161400
H	-7.67753500	0.90599100	3.15508300
H	-7.58977200	-0.78817200	2.61748700
H	-8.50803100	0.41393000	1.68067300
H	-5.64815900	-1.28226400	1.64257500
C	0.06685600	2.84570700	-1.33414900
H	0.14987900	3.82276500	-1.83112600
H	-0.68930900	2.96556300	-0.54995900
C	1.83930700	3.54934600	0.34785400
H	1.09219700	3.56732900	1.15359600
H	1.78164400	4.53845600	-0.12965100
C	3.42733600	2.23587300	1.95964500
H	4.23764600	2.49682100	2.65567600
H	2.52357300	2.14906100	2.57929100
C	3.94386000	-0.19268300	2.47781500
H	3.01919100	-0.25137700	3.06933600
H	4.70956900	0.19114500	3.16736300
C	3.20688300	-2.54970800	1.61203200
H	3.60192900	-3.57554400	1.57105400
H	2.43690700	-2.55924700	2.39619000
C	1.53369800	-3.34572900	-0.12665400
H	0.83463300	-3.49216900	0.70820600
H	2.07277500	-4.29653900	-0.24336600
H	2.98333300	0.54297400	0.66695100
H	2.18732600	2.40889400	-1.46330400
H	3.29032300	-2.14093400	-0.51636900
